# Interleukin-11 Drives Early Lung Inflammation during *Mycobacterium tuberculosis* Infection in Genetically Susceptible Mice

**DOI:** 10.1371/journal.pone.0021878

**Published:** 2011-07-15

**Authors:** Marina A. Kapina, Galina S. Shepelkova, Vadim G. Avdeenko, Anna N. Guseva, Tatiana K. Kondratieva, Vladimir V. Evstifeev, Alexander S. Apt

**Affiliations:** Department of Immunology, Central Institute for Tuberculosis, Moscow, Russia; Fundação Oswaldo Cruz, Brazil

## Abstract

IL-11 is multifunctional cytokine whose physiological role in the lungs during pulmonary tuberculosis (TB) is poorly understood. Here, using *in vivo* administration of specific antibodies against IL-11, we demonstrate for the first time that blocking IL-11 diminishes histopathology and neutrophilic infiltration of the lung tissue in TB-infected genetically susceptible mice. Antibody treatment decreased the pulmonary levels of IL-11 and other key inflammatory cytokines not belonging to the Th1 axis, and down-regulated IL-11 mRNA expression. This suggests the existence of a positive feedback loop at the transcriptional level, which is further supported by up-regulation of IL-11 mRNA expression in the presence of rIL-11 in *in vitro* cultures of lung cells. These findings imply a pathogenic role for IL-11 during the early phase of *Mycobacterium tuberculosis*-triggered disease in a genetically susceptible host.

## Introduction

IL-11 is multifunctional cytokine with hematopoietic, immunomodulatory, and epithelial cell protective activities [1]. It is a member of the IL-6 cytokine family which includes cytokines that share the use of the gp130 molecule in their receptor complexes [2], [3]. In several clinical and experimental studies, IL-11 displayed anti-inflammatory activity [4]–[7], although its over-expression may have a significant pro-inflammatory effect [8]. IL-11 augmented bone marrow recovery and platelet production, down-regulated pro-inflammatory type 1 cytokines, and has been approved for the human use for corresponding indications [1], [9], [10]. In the lungs, IL-11 was shown to be produced by epithelial cells, fibroblasts, smooth muscle cells and antigen-presenting cells in response to different stimuli, including respiratory viruses [11]–[14]. Using the ovalbumin-induced asthma model in transgenic and IL-11Rα-disrupted mice, it was demonstrated that IL-11 is involved in regulation of such Th2-type pulmonary responses as eosinophilic inflammation, mucus metaplasia and IL-13 production, without shifting the response towards Th1 inflammation [15]–[17].

The role of IL-11 during pulmonary tuberculosis (TB) and accompanying lung inflammation is poorly understood. *In vitro* studies from our laboratory demonstrated that the important producers of IL-11 are interstitial lung macrophages, and that the level of mRNA for IL-11 in these cells differs substantially between mouse strains, being higher in TB-susceptible I/St compared to TB-resistant A/Sn mice [18]. More recently, using infected (I/St×A/Sn) F2 hybrids segregating for the level of TB severity, it was demonstrated that the individual levels of IL-11 mRNA in the lung tissue correlated inversely with rapid body weight loss, the phenotype characteristic for *Mycobacterium tuberculosis*-triggered disease [19]. However, there was no direct physiologic evidence that disease progression and immune responses in the lungs could be altered by manipulating IL-11 production *in vivo*. Here, we show that blocking endogenous IL-11 with specific antibodies *in vivo* attenuates the severity of TB in genetically susceptible I/St mice. Moreover, we demonstrate that antibody treatment not only decreases the lung IL-11 content, but also down-regulates its mRNA expression, suggesting the existence of a positive feed-back loop at the transcriptional level, which is supported by *in vitro* experiments.

## Results and Discussion

### Rapid IL-11 response in the lungs of genetically susceptible mice after TB challenge and therapeutic effect of the anti-IL-11 treatment

**Figure 1:**
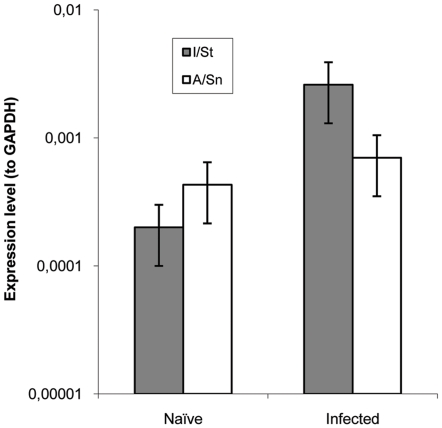
Two weeks after TB challenge the level of IL-11 mRNA increases ∼1 log in the lungs of TB-susceptible I/St but does not change in TB-resistant A/Sn mice. Mean ± SEM expression level plotted against that of GABDT in 4 individual mice per group is displayed (*P*<0.01, ANOVA, between naïve and infected I/St mice).

Earlier we found that isolated and cultured interstitial lung macrophages from TB-susceptible I/St mice produced significantly more IL-11 than their counterparts from TB-resistant A/Sn mice [18]. Since numerous cell types are capable of producing this cytokine in the lungs [11]–[14], [18], it was useful to evaluate whether or not TB-susceptible and resistant mice differed in the expression of IL-11 *in vivo* at the whole-organ level before and after TB infection. Assessment of mRNA extracted from the whole lungs of mice of the two strains by DNA microarray provided a ∼5-fold increase (2^ΔCt^ = 2.3) in *il11* expression in TB-infected compared to naïve I/St mice, whereas its expression in A/Sn mice did not change after TB challenge (2^ΔCt^ = 0.7). To address this issue more precisely, we compared the expression level of the *il11* gene in the lungs before and after TB challenge using qrt-PCR. At the whole-organ level, naïve A/Sn mice produced slightly more IL-11 mRNA compared to naïve I/St mice, which may reflect its production by cells other than lung macrophages and/or the difference between *in vivo* and *in vitro* systems. However, at 2 weeks post challenge, the levels of IL-11 mRNA remained the same in the lungs of A/Sn mice, but increased ∼10-fold (*P*<0.01) in I/St mice (Fig. 1), suggesting an altered control of the infection-induced early IL-11 production in genetically susceptible animals. Importantly, the different kinetics of *il11* expression in the two mouse strains can not be explained by a more rapid accumulation of mycobacteria (stimulus) in the lungs of I/St mice, since there is no difference in mycobacterial growth between I/St and A/Sn mice until 3 weeks post challenge ([20], confirmed in this study, data not shown). It is also unlikely that a rapid increase in IL-11 response is due to some specific features of I/St genetic background: a reverse correlation between the level of IL-11 expression in the lungs and severity of early TB was demonstrated in a big segregating population of (I/St×A/Sn) F2 mice with highly diverse individual genetic compositions [19]. These observations prompted us to perform blocking experiments in an attempt to diminish the severity of the TB course in I/St mice.

**Figure 2:**
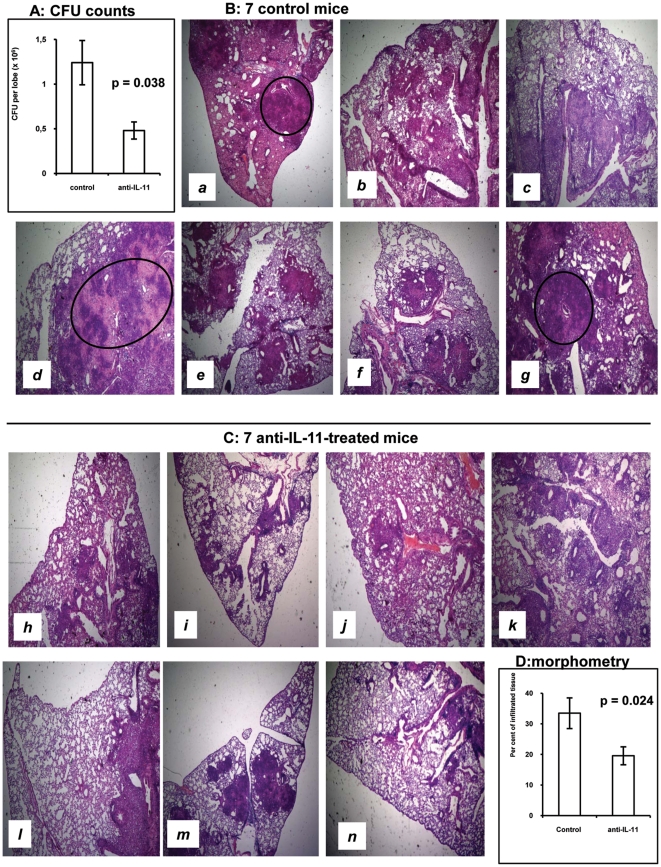
Treatment with anti-IL-11 antibodies significantly attenuates the severity of TB in I/St mice. (A) ∼3-fold decrease in lung CFU counts compared to control animals. (B and C) Lung pathology in individual animals. None of anti-IL-11-treated mice developed necrotic TB foci evident in control mice *a*, *d* and *g* (circled). (D) Statistical evaluation of the proportion of inflamed lung tissue. CFU counts and morphometry were performed in all mice included in 2 independent experiments (total N = 16 and 17 for experimental and control groups, respectively). Histology is displayed for individual mice analyzed in one experiment (N = 7 for each group).

Groups of I/St mice were infected and treated with either anti-IL-11 antibodies or pre-immune globulin as described in Materials & Methods, and mycobacterial loads in the lungs were compared between groups at day 24 post challenge. As shown in Fig. 2A, significantly fewer CFU were recovered from the lungs of anti-IL-11-treated mice, indicating a beneficial effect of treatment. We also compared the severity of lung pathology between experimental and control groups and found that anti-IL-11-treated animals did not develop necrotizing and/or coalescing TB foci (Fig. 2C), which were readily detected in a proportion of control animals (Fig. 2B). This is an important observation, since both in humans and animals areas of necrosis and surrounding acellular matrix (rim structure) are primary sites for production of large numbers of bacteria [21], [22], which provides a good explanation for the difference in CFU counts. A quantitative evaluation of pathology demonstrated that significantly smaller areas of the lung tissue were affected by inflammation in the experimental compared to the control group (Fig. 2D).

Taken together, these results clearly demonstrate a detrimental effect of the early IL-11 hyper-production in response to mycobacteria and suggest its causative role in pathogenesis of *M. tuberculosis*-triggered lung disease in mice.

### Cellular infiltration and immune responses in the lungs

To find out how anti-IL-11 treatment alters infection-induced cellular infiltration, we assessed the content of major immune cell types in the lungs of infected mice. As shown in Table 1, the only statistically significant difference between mice that received non-immune rabbit globulin and anti-IL-11 antibodies was a reduced neutrophil content in the latter group. There is ample evidence that neutrophilic inflammation plays a detrimental role in mycobacteria-induced pathology. We and others, using genetic approaches, demonstrated deleterious rather than beneficial effects of these early inflammatory cells in the course of chronic mycobacterial infections [23]–[26]. The data presented herein add to this line of evidence, demonstrating the role of IL-11 in neutrophilia and providing an explanation for an early appearance of necrotic zones in the lungs of control mice displayed in Fig. 1B. As the granulomata mature, lung-infiltrating neutrophils die rapidly and the sites of their accumulation are replaced by necrotic zones [24].

**Table 1:** Administration of anti-IL-11 antibodies decreases neutrophil influx in the lungs of TB-infected mice*.

Group	Cellular composition^a^										
	Total cell count	CD4^+^		CD8^+^		CD19^+^		F4/80^+^		Ly-6G^+^	
		Per cent	10^6^/lobe	Per cent	10^6^/lobe	Per cent	10^6^/lobe	Per cent	10^6^/lobe	Per cent	10^6^/lobe
Control Ig	15.1±1.5	40.6±2.4	6.1±0.5	19.6±1.8	3.0±0.2	14.1±1.2	2.2±0.2	8.7±0.6	1.3±0.2	**15.2±2.5**	**2.3±0.3**
Anti-IL-11	12.8±1.3	40.7±2.3	5.2±0.5	19.4±1.2	2.5±0.4	19.9±2.6	2.6±0.3	9.1±0.6	1.2±0.1	**8.4±1.2**	**1.1±0.2**
*P*	0.278	>0.7		>0.5		0.384		>0.8		**0.024** b	

*Middle right lobes were individually isolated from two groups of mice (two independent experiments, exp. 1: N = 7 and 7; exp. 2: N = 9 and 10; total N = 16_control_ and N = 17_exp_.), enzymatically disrupted, and single-cell suspensions analyzed by flow cytometry using FITC- or PE-labeled mAbs to the indicated surface markers. Results are presented as summarized mean ± SD for all animals.

a For F4/80^+^ macrophages and Ly-6G^+^ PMN: per cent of the total cell count; for B- and T-lymphocytes: per cent of the population gated for the lymphocyte size. Significant (Student's *t*-test) difference in the neutrophil content between experimental and control groups is highlighted in bold.

b The only statistically significant difference between mice that received control rabbit globulin and anti-IL-11 antibodies was a decrease in the lung neutrophil infiltration in the latter group.

**Figure 3:**
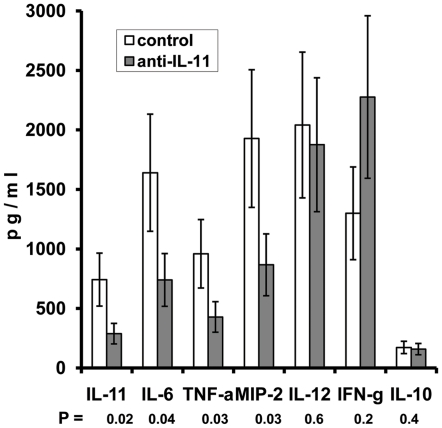
Anti-IL-11 antibody therapy decreases levels of IL-11 and pro-inflammatory factors in the lung tissue without shifting the IL-12 – IFN-γ/IL-10 balance. Cytokine contents in lung homogenates were assessed by ELISA for 4 mice in each group. The results of one of two similar experiments are displayed as mean ± SEM.

We also assessed how the blocking of IL-11 influences the production of major cytokines in the lungs of infected mice. Treatment with anti-IL-11 antibodies significantly decreased the level of IL-11 itself, as well as the levels of key pro-inflammatory and immunoregulatory molecules – IL-6, TNF-α and MIP-2. On the other hand, the Th1-shifted immune response was not affected by the antibody administration: the prominent production of IL-12 and IFN-γ and the marginal to un-measurable production of IL-10 and IL-4 (not shown) was equal in antibody-treated and control mice (Fig. 3).

The question about IL-11 involvement in Th1/Th2 modulation remains unresolved. For example, there is evidence in cell culture systems that IL-11 can down-regulate IL-12 and IFN-γ production [27], [28], but *in vivo* studies in gene knock-out and transgenic mice demonstrated its capacity to down-regulate Th2 cytokine production [11], [17]. It is difficult to judge which activity, if any, might predominate during natural, un-manipulated *in vivo* conditions. The results of our blocking experiments in the mouse model of pulmonary TB clearly demonstrate that IL-11 substantially promotes lung inflammation but does not inhibit the Th1 response. It is quite possible that there is no general physiological pattern of immune response regulation by IL-11, and that it is dependent upon the biological context within which the effects of IL-11 are assessed.

### Autocrine regulation of IL-11 production at the transcriptional level

**Figure 4:**
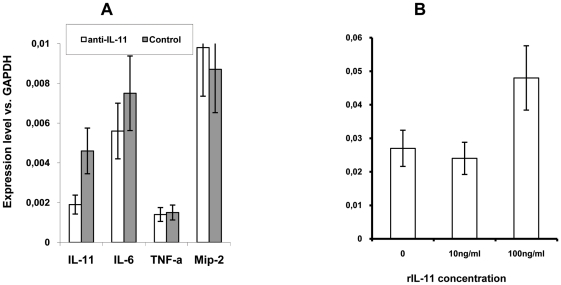
Protein levels of IL-11 affect IL-11 mRNA expression. (A) *In vivo* administration of anti-IL-11 antibodies leads to a selective down-regulation of IL-11 mRNA. The level of expression was quantified in 5 individual mice per group, using qrt-PCR and normalization against the level of GAPDH expression. Results obtained in 1 of 2 similar experiments are expressed as mean ± SEM (for IL-11 expression *P* = 0.021, for other cytokines *P*>0.05). (B) Introduction of 100 ng/ml rIL-11 in cultures of lung cells up-regulates the expression of IL-11 mRNA. Results of two similar experiments are expressed as mean of 3 wells ± SEM (*P*<0.01, ANOVA, compared to negative controls and cultures stimulated with 10 ng/ml IL-11).

Our data demonstrate that elevated lung IL-11 production starts very early after infection, and that neutralization of IL-11 diminishes both lung pathology and production of key regulatory cytokines. To find out whether a decrease in pulmonary IL-11, IL-6, TNF-α and MIP-2 protein production at day 24 post challenge was due to down-regulation of the expression of the corresponding genes, we assessed the levels of *il11*, *il6*, *tnfa* and *mip2* mRNA in the lung tissue of mice treated with either anti-IL-11 antibodies or control globulin. To our surprise, the only factor whose lung mRNA expression significantly decreased in anti-IL-11 treated mice was IL-11 itself (Fig. 4A). This result suggested that IL-11 expression is regulated post-infection in an autocrine manner at the translational level. On the other hand, no significant changes at the mRNA expression level were observed for IL-6, TNF-α and MIP-2, indicating that their decreased production in anti-IL-11-treated mice may well be due to reduced inflammation compared to control animals. To test the existence of a positive feed back loop, we assessed the level of IL-11 mRNA in lung cells cultured in the presence or absence of recombinant murine IL-11. As shown in Fig. 4B, 12-h incubation of lung cells in the presence of 100 ng/ml of rmIL-11 resulted in a significant increase in the *il11* mRNA levels compared to control cells, supporting our hypothesis.

Overall, our findings suggest that IL-11 production is rapidly elevated and self-supported in the lungs of genetically susceptible TB-infected mice. This promotes the development of early inflammation with a substantial contribution by neutrophils which, in turn, biases the response towards necrotic granuloma formation, accompanied by accelerated mycobacterial multiplication and a “cytokine storm” [29], thus, exacerbating pulmonary TB pathology.

## Materials and Methods

### Animals

Female mice of the I/StSnEgYCit strain (hereafter – I/St) aged 9–10 weeks were used. Compared to several inbred mouse strains, including A/SnEgCit (A/Sn) also used in this study, these mice display an extremely high level of susceptibility to TB, severity of lung pathology and prominent infiltration of *M. tuberculosis*-infected lung tissue with neutrophils, regardless to the dose and route of challenge [23], [24], [30], [31]. Mice were bred under conventional conditions at the Animal Facilities of the Central Institute for Tuberculosis (CIT, Moscow, Russia), in accordance with the guidelines from the Russian Ministry of Health # 755, NIH Office of Laboratory Animal Welfare (OLAW) Assurance #A5502. Water and food were provided *ad libitum*. All experimental procedures were approved by the CIT animal care committee (IACUC protocols #2, 7, 8, 11, 13 approved on September 20, 2009).

### Anti-IL-11 antibodies

Two conventional white rabbits were immunized with 0.05 µg of recombinant murine (rm) IL-11 (Sigma, St-Louis, MA) in incomplete Freund's adjuvant (Sigma), thrice, subcutaneously, with a 3-wk interval between injections. Before the first immunization, animals were bled and their serum immunoglobulin was affinity purified and depleted of anti-mycobacterial activity using procedures identical to those described below for immune sera. The mixed globulin preparation obtained from two non-immune rabbits served as the autologous control for *in vivo* administration. One month after the third immunization, animals were bled, and the level of anti-IL-11 antibodies in their mixed sera was assessed by indirect ELISA using rmIL-11 and immune anti-rabbit IgG conjugate (Bio-Rad, Richmond, CA). Serum IgG was isolated using Sepharose CL4B-protein A adsorbent (Pharmacia, Uppsala, Sweden).

**Figure 5:**
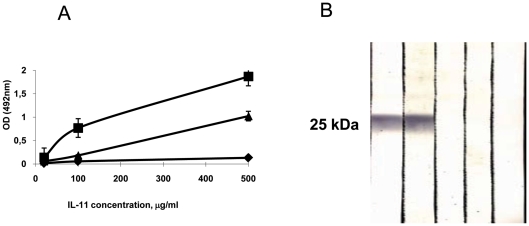
Properties of anti-IL-11 polyclonal antibodies. (A) Reactivity of affinity purified rabbit globulin preparation against mIL-11 assessed in ELISA format. No reactivity was found in pre-immune globulin (diamonds); immunoglobulin from rabbits immunized thrice with rmIL-11 showed very strong reactivity (squares); after exhaustion on mycobacterial sonicate adsorbent, specific anti-IL-11 reactivity dropped but was readily detected (triangles). (B) Immune blotting with rmIL-11 with polyclonal rabbit anti-mIL-11 antibodies (preparation identical to one displayed as triangles in (A). Tracks: 1, 2 – immune rabbits 1 and 2; 3, 4 – pre-immune rabbits 1 and 2; 5 – conjugate-free control.

Normal rabbit immunoglobulin possesses an intrinsic anti-mycobacterial activity which, after *in vivo* administration, could modulate the course of mycobacterial infection [32]. To avoid interference with their specific anti-IL-11 activity, immune and non-immune preparations were depleted of anti-mycobacterial activity using affinity chromatography. To this end, we prepared an immune adsorbent of *Mycobacterium tuberculosis* H37Rv sonicate bound to BrCN-activated Sepharose CL6B (Pharmacia, Uppsala, Sweden), as described [33], and repeatedly absorbed immunoglobulin preparations until the activity against mycobacterial sonicate assessed by immune blotting was completely lost (data not shown). Reactivity of the control and anti-IL-11 immunoglobulin preparations was assessed in the ELISA format and by immune blotting using rmIL-11 (Fig. 5A, B).

### Administration of antibodies and infection

At day 0, mice were infected with 10^3^ CFU *Mycobacterium tuberculosis* H37Rv (Pasteur) via intra-tracheal route exactly as described previously [31]. At days −1, +2, +14, +17, +20 and +22 mice were injected peritoneally with 50 µg/mouse of either anti-IL-11 (experiment) or pre-immune (control) purified rabbit immunoglobulin preparation. Two independent experiments in groups of 7–10 control and experimental animals each provided almost identical results, which were combined for the statistical analysis of the major phenotypes.

### CFU counts

Mice were sacrificed at day 24 post-challenge. Apical right lobes from individual mice (in two experiments total N = 17_contr_. and N = 18_exp_.) were homogenized in 1 ml of sterile saline, and 0.1 ml of serial 10-fold dilutions of homogenates were plated onto Dubos agar. Colonies were counted after 18–20 days of incubation at 37°C.

### Histopathology

Left lungs of individual mice were removed, inflated with 4% formalin in PBS (pH = 7.2) via the bronchus, and fixed in 4% formalin for 24 h. The samples were processed, embedded in paraffin, sectioned and stained with hematoxylin and eosin. Morphometry was performed at the ×37.5 magnification using Axioskop 40 microscope and AxioVision 4.8.1.0 software (Carl Zeiss, Berlin, Germany). The area of inflamed lung parenchyma, excluding vessels and airways, was measured and calculated as a percentage of the area of total lung parenchyma.

### Cytokine mRNA quantification

Total RNA from the lower right lung lobes of individual mice was isolated using the commercial SV Total RNA Isolation System, and reverse transcription of mRNA was performed using reagents and protocols from Promega, Madison, WI. To detect the mRNA levels for a number of inflammation-related genes, quantitative real-time RT-PCR (qrt-PCR) with cDNA was performed using the iCycler iQ Multicolor Real-Time PCR Detection System (BioRad, Hercules, CA), and specific primers, TaqMan probes and reagents from Applied Biosystems (Foster City, CA). Gene expression levels in the lung tissue of individual mice were normalized to those of GAPDH. To quantify the results obtained by real-time PCR, the comparative threshold method was used exactly as described in [34], with the expression of the results as mean fold increase ± SEM for groups of 4 mice each.

To assess the ability of rIL-11 to up-regulate its own mRNA production, lung cell suspensions were prepared as described in [31], and cultured in RPMI-1640 medium containing 5% FCS and standard supplements (all components – HiClone, HiClone, Logan, UT, USA). Lung cells were cultured in 6-well plates (Costar, Badhoevedorp, The Netherlands) at 1.5×10^6^ cells/ml in the absence or presence of 10 ng/ml and 100 ng/ml rmIL-11 (ImmunoTools, Friesoythe, Germany) for 12 h before RNA was isolated, reversely transcribed, and *il11* expression was assessed with normalization to that of GAPDH. Two independent experiments, performed with mixtures of RNA from 3 I/St mice each, provided similar results.

### Flow cytometry and cytokine ELISA

Middle right lobes were individually isolated, enzymatically disrupted as described in [31], and single-cell suspensions analyzed by flow cytometry using FITC- or PE-labeled mAbs to the indicated surface markers (Table 1). Results are presented as summarized mean ± SEM for all animals.

To measure cytokine contents, the rest of each sample was centrifuged, and the pellets frozen at −70°C until assessed. The thawed samples from 4 mice in each group were diluted in 2 ml of PBS, debris removed by centrifugation at 500 g, and the cytokine content assessed in ELISA format using the ELISA kits for IL-6, IL-10, IL-12, MIP-2, TNF-α and IFN-γ purchased from PharMingen, San-Diego, CA, and for IL-11 purchased from R&D Systems, Minneapolis, MI, according to the manufacturers' instructions.

### Statistical Analyses

One-tail analysis of variance (ANOVA) and Student's *t*-test were used as indicated in figure legends and table footnotes. *P*<0.05 was considered statistically significant.
